# Anxiety and Its Influencing Factors in Patients With Drug-Induced Liver Injury

**DOI:** 10.3389/fpsyg.2022.889487

**Published:** 2022-06-28

**Authors:** Yi-Hui Liu, Yan Guo, Hong Xu, Hui Feng, Dong-Ya Chen

**Affiliations:** ^1^Department of Digestive Hepatology, Hangzhou Red Cross Hospital, Hangzhou, China; ^2^Department of Gastroenterology, Hangzhou Third Hospital, Hangzhou, China

**Keywords:** drug-induced liver injury, anxiety, influencing factors, type of liver injury, degree of liver injury

## Abstract

**Objective:**

This study aims to investigate anxiety and its influencing factors in patients with drug-induced liver injury (DILI).

**Materials and Methods:**

Ninety-four patients with DILI were enrolled and evaluated with a self-rating anxiety scale (SAS). According to the anxiety score, they were divided into four groups: the non-anxiety, mild anxiety, moderate anxiety, or severe anxiety groups, and the scores were analyzed based on demographic and biochemical indicators.

**Results:**

Of the 94 patients with DILI, 63 did not have anxiety and 31 had anxiety (32.9%), of which 27 had mild, 3 had moderate, and 1 had severe anxiety. There were no statistically significant differences in gender, age, occupation, and level of education between the groups (*F* = 1.42, *H* = 2.361, *H* = 6.751, *H* = 1.796, and *P* > 0.05); anxiety score and degree of anxiety between the types of drugs that led to the liver injury (*H* = 0.812, *H* = 1.712, and *P* > 0.05); anxiety score between the different degrees of liver injury (*H* = 2.836, *H* = 4.957, *P* > 0.05); or length of hospital stay or prognosis between the degrees of anxiety (*F* = 1.487, *H* = 0.761, *P* > 0.05). However, there were statistically significant differences in the degree of anxiety between different degree and types of liver injury (*H* = 7.981, *H* = 8.208, *P* < 0.05).

**Conclusion:**

Patients with DILI may have anxiety, especially mild anxiety. The occurrence of anxiety in patients with DILI is not related to gender, age, occupation, or level of education but may be related to the degree and type of liver injury. Anxiety has no impact on the length of stay in hospital or the prognosis of the DILI. These findings may contribute to the development of management strategies for patients with DILI.

## Introduction

Studies have revealed that chronic liver disease is closely related to anxiety and depression, and psychological factors affect the development and prognosis of patients with chronic liver disease, such as chronic hepatitis B and liver cirrhosis (Popović et al., 2015; Buganza-Torio et al., 2019), but there are few studies on anxiety and depression in patients with acute liver disease. In recent years, the rate of incidence of drug-induced liver injury (DILI) has been increasing, but studies on the correlation between DILI and anxiety are rare. According to our clinical experiences, patients with DILI may still have anxiety despite a good prognosis due to the lack of medical knowledge, personal stress resistance, and other reasons, which may significantly affect the treatment of the disease. However, few studies have focused on this area. Therefore, preliminary observations were carried out in this study, and the details are reported as follows.

## Materials and Methods

### Subjects

A total of 94 patients with DILI treated in our department between January 2019 and June 2020 were enrolled. All patients were hospitalized for liver diseases. This study was approved by the Academic Ethics Committee of the Hangzhou Red Cross Hospital. All patients signed an informed consent.

### Inclusion Criteria

According to the 2015 guidelines for the diagnosis and treatment of DILI (Pharmaceutical Liver Disease Group, Liver Disease Branch, Chinese Medical Association, 2015), the Roussel Uclaf Causality Assessment Method score was >6 points (Danan and Teschke, 2016), the patients passed the updated RUCAM assessment and met the classification standard for DILI.

### Exclusion Criteria

(1) Patients complicated with hepatitis A, B, C, or E or human immunodeficiency virus infections; (2) patients complicated with drug-induced, fatty, alcoholic, and genetic metabolic liver disease; decompensated chronic liver disease; or other autoimmune diseases; (3) pregnant or lactating women; (4) patients with major diseases of the cardiovascular, cerebrovascular, or hematopoietic system, liver, or kidney; or (5) patients with a history of mental illness such as anxiety or depression.

### Research Methods

The patients’ age, gender, occupation, level of education, serum liver function including total bilirubin (TBIL), alanine aminotransferase (ALT), glutamic oxaloacetic transaminase (AST), alkaline phosphatase (ALP), and gamma-glutamyl transferase were recorded. The upper limit of normal (ULN) for TBIL is 3–20.5 μmol/L, for ALT is 9–50 U/L, for AST is 13–35 U/L, and for ALP is 45–125 U/L. The self-rating anxiety scale (SAS) (Zung, 1971) was used to evaluate the patients’ degree of anxiety and has a total of 20 items, which are ranked on a scale of 1–4 points. Therefore, the highest cumulative raw score is 80, which is then multiplied by 1.25 to convert it into a standard total score. An SAS standard total score of >50 points indicates that the patient exhibits symptoms of anxiety and is evaluated as suffering from anxiety with 50–59 points indicating mild, 60–69 moderate, and >69 severe anxiety. The SAS score was obtained from each subject immediately after enrollment.

### Determination of the Type of Liver Injury

Drug-induced liver injury was classified according to the 2015 edition of the guidelines for the diagnosis and treatment of DILI (Pharmaceutical Liver Disease Group, Liver Disease Branch, Chinese Medical Association, 2015): (1) hepatocyte injury type: ALT ≥ 3 × ULN and R ≥ 5; (2) cholestasis type: ALP ≥ 2 × ULN and R ≤ 2; and (3) mixed type: ALT ≥ 3 × ULN, ALP ≥ 2 × ULN, and 2 < R < 5 (*R* = [ALT ÷ ULN] ÷ [ALP ÷ ULN]).

### Determination of the Degree of Liver Injury

The degree of liver injury was also determined using the 2015 edition of the guidelines for the diagnosis and treatment of DILI (Pharmaceutical Liver Disease Group, Liver Disease Branch, Chinese Medical Association, 2015): (1) grade 1 (mild liver injury): serum ALT and/or ALP showed a recoverable increase, TBIL < 2.5 × ULN; (2) grade 2 (moderate liver injury): serum ALT and/or ALP increased, TBIL ≥ 2.5 × ULN; and (3) grade 3 (severe liver injury): serum ALT and/or ALP increased, TBIL ≥ 5 × ULN.

### Criteria for Curative Effect

The patient was considered to be cured if TBIL, ALT, and AST all returned to normal levels. The patient was considered to be improved if TBIL, ALT, and/or AST improved after treatment but did not return to normal levels. The treatment was considered to be ineffective if TBIL, ALT, and AST did not improve.

### Statistical Methods

Data were processed using the statistical analysis software SPSS 26.0. The data of normal distribution were described by mean ± standard deviation, while those of non-normal distribution were represented as median (quartile range, Q_*R*_). Multi-group comparison of normally distributed data was conducted using one-way analysis of variance, while that of count data or non-normally distributed data was conducted using the rank sum and Kruskal–Wallis tests. *P* < 0.05 was considered to be statistically significant.

## Results

### General Data

Of the 94 patients with DILI who were enrolled in the study, 23 were males and 71 patients were females. All patients passed the updated RUCAM assessment, and RUCAM score: 65 cases with 6–8 points (very likely), 29 cases with ≥9 points (most likely). The average age of the patients was 54.54 ± 15.84 years old, and the group consisted of 17 farmers, 12 workers, 13 employees, and 52 people in other occupations (retired/unemployed/free). The levels of education were divided into junior high school and below (*n* = 60), senior high school (*n* = 18), and junior college and above (16). The following associated diseases were observed in this cohort: tuberculosis (17 cases), bacterial infection (9 cases), dermatitis (9 cases), insomnia (6 cases), tumors (5 cases), upper respiratory tract infections (5 cases), Joint injury (4 cases), constipation (3 cases), hyperlipidemia (3 cases), chronic gastritis (5 cases), menstrual disorders (6 cases), others (22 cases). The average anxiety score of the patients was 43.01 ± 10.36. In terms of the degree of anxiety, 63 patients had no anxiety, 27 had mild anxiety, 3 had moderate anxiety, and 1 had severe anxiety.

### Demographic Characteristics of Patients With Drug-Induced Liver Injury

The differences in gender, age, occupation, and level of education between the four groups were not statistically significant (*P* > 0.05, see Table 1).

**TABLE 1:** General characteristics of DILI patients with anxiety.

Anxiety degree	*n*	Gender (male/female)	Age (years)	Occupation				Level of education		
					
				Farmer	Worker	Staff member	Others	Junior high school and below	High school	Junior college or above
Non	63	18/45	53.30 ± 16.15	11	11	12	29	41	12	10
Mild	27	5/22	58.41 ± 14.94	6	1	1	19	17	5	5
Moderate	3	0/3	42.33 ± 12.58	0	0	0	3	1	1	1
Severe	1	0/1	65.00	0	0	0	1	1	0	0
*F*-value (3,90)			1.42							
*H*-value		2.361		6.751					1.796	
*P*-value		0.501	0.24	0.08					0.616	

*F-value indicates test statistic for ANOVA. H-value indicates test statistic for Kruskal–Wallis test.*

### The Influence of the Type of Drug That Led Liver Injury on Anxiety

The categorical types of drugs that caused liver injury included Western medicine (anti-tuberculosis drugs, antibiotics, chemotherapy drugs, anti-inflammatory painkillers, hypolipidemic drugs, acid-suppressing drugs, etc.), traditional Chinese medicine/Chinese patent medicine (Chinese medicine compound decoctions, granules/proprietary Chinese medicines), and healthcare supplements (dietary medicines). The differences in anxiety score and degree of anxiety between the different types of drugs that led to liver injury were not statistically significant (*P* > 0.05, see Table 2).

**TABLE 2:** Influence of drugs leading to liver injury on anxiety.

Drug	*n*	Anxiety score median (Q_*R*_)	Anxiety degree			
			
			Non	Mild	Moderate	Severe
Western medicine	42	40.5 (18.8)	27	12	3	0
Traditional Chinese medicine/ Chinese patent medicine	44	41.0 (14.5)	32	11	0	1
Healthcare supplements	8	46.5 (14.5)	4	4	0	0
*H*-value		0.812			1.712	
*P*-value		0.666			0.425	

*H-value indicates test statistic for Kruskal–Wallis test.*

### Relationship Between the Degree of Liver Injury and the Degree of Anxiety

The difference in anxiety score between the three groups based on the degree of liver injury was not statistically significant (*P* > 0.05). However, the degree of anxiety in patients with mild, moderate, and severe livery injury was statistically significant (*P* < 0.05, see Table 3).

**TABLE 3:** Relationship between the degree of liver injury and the degree of anxiety.

Degree of liver injury	*n*	Anxiety score median (Q_*R*_)	Anxiety degree			
			
			Non	Mild	Moderate	Severe
Mild	61	45.0 (18.0)	35	22	3	1
Moderate	12	39.5 (10.0)	11	1	0	0
Severe	21	41.0 (13.0)	17	4	0	0
*H*-value		2.836			7.981	
*P*-value		0.242			0.018	

*H-value indicates test statistic for Kruskal–Wallis test.*

### Relationship Between the Type of Liver Injury and the Degree of Anxiety

The difference in anxiety score between the three groups based on the types of liver injury was not statistically significant (*P* > 0.05), while the difference in degree of anxiety was significant (*P* < 0.05, see Table 4).

**TABLE 4:** Relationship between the type of liver injury and the degree of anxiety.

Type of liver injury	*n*	Anxiety score median (Q_*R*_)	Anxiety degree			
			
			Non	Mild	Moderate	Severe
Hepatocyte type	85	41.0 (16.5)	60	23	2	0
Cholestasis type	5	54.0 (23.0)	1	2	1	1
Mixed type	4	44.0 (20.0)	2	2	0	0
*H*-value		4.957			8.208	
*P*-value		0.084			0.017	

*H-value indicates test statistic for Kruskal–Wallis test.*

### The Effect of Anxiety on Recovery From Drug-Induced Liver Injury

The differences in the length of hospital stay and prognosis between the groups were not statistically significant (*P* > 0.05, see Table 5).

**TABLE 5:** Effect of anxiety on the recovery of DILI.

Anxiety degree	*n*	Hospitalization stay (days)	Prognosis		
			
			Cured	Improvement	Ineffective
Non	63	15.41 ± 8.08	23	40	0
Mild	27	12.11 ± 5.03	11	16	0
Moderate	3	12.67 ± 3.06	1	2	0
Severe	1	10 ± 0.00	0	1	0
*F*-value (3,90)		1.487			
*H*-value				0.761	
*P*-value		0.224		0.859	

*F-value indicates test statistic for ANOVA. H-value indicates test statistic for Kruskal–Wallis test.*

## Discussion

Previous studies have focused on the relationship between chronic liver disease and it is believed that the severity of illness is related to anxiety and depression, which may in turn affect the outcome of the disease. Banerjee et al. (2020) revealed that chronic liver disease with anxiety and depression seriously affects quality of life for patients. It has been reported that female gender, low levels of education, self-financing, and low family income were factors influencing anxiety and depression in patients with chronic hepatitis B (Zheng et al., 2020). Labenz et al. (2020) revealed that non-alcoholic fatty liver disease (NAFLD) is an independent risk factor for anxiety and depression. Also, patients with NAFLD had were significantly higher rates of anxiety and depression compared with healthy subjects (Tao et al., 2017). Women with NAFLD are more likely to develop anxiety and depression than men and had a higher incidence of steatosis (Choi et al., 2021). The study by Zhang et al. (2020) found that the feeling of being discriminated against in daily life was the main influencing factor for anxiety and depression in patients with acute hepatitis B. Anxiety and depression were common in acute hepatitis C infection, but it was not related to the severity of the disease, the method of infection, or the rate at which patients were lost to follow-up (Deterding et al., 2016). Even for high-risk patients with severe mental disorders, direct antiviral therapy had no effect on anxiety or depression during or after treatment for chronic hepatitis C infection (Gallach et al., 2018). Kong et al. (2021) showed that the incidence of anxiety increased in patients with chronic hepatitis B treated with oral antiviral therapy, and female gender and length of treatment were high risk factors. The incidence of anxiety and depression in patients with autoimmune hepatitis is also very high (Schramm et al., 2014; Janik et al., 2019). The severity of cirrhosis and the Child-Pugh score are positively correlated with anxiety and depression (Wu et al., 2020). Moreover, one out of six patients with cirrhosis have moderate to severe depression and nearly half have moderate to severe anxiety (Hernaez et al., 2020). Xiao et al. (2018) revealed that patients with hepatitis B cirrhosis have sleep disorders, which may be a symptom of mild hepatic encephalopathy that may cause more serious anxiety. At present, few studies have investigated whether DILI may lead to anxiety and its influencing factors. Our study provided preliminary findings on this topic.

As a result of increased attention being paid to health and the widespread use of drugs and healthcare products, the incidence of DILI is increasing, and most cases are acute. DILI has become one of the most important causes of acute liver injury. In the treatment of underlying diseases, DILI may aggravate negative emotions and even affect the treatment, especially in patients with chronic diseases, such as cancer and tuberculosis. However, there are few reports investigating anxiety in patients with DILI, and its incidence and influencing factors are unknown. This study revealed that of the 94 patients with DILI, 31 had anxiety (32.9%), with mild anxiety being the most common, while moderate to severe anxiety was rare. This result suggests that like chronic liver disease, DILI can also lead to anxiety.

In terms of treatment, in addition to drugs, we should pay attention to psychological counseling and ensure that patients are educated and equipped with disease-related knowledge. Many studies conclude that female gender and advanced age are high risk factors for DILI (Pharmaceutical Liver Disease Group, Liver Disease Branch, Chinese Medical Association, 2015; George et al., 2018; Shen et al., 2019). The degree of anxiety and depression in patients with chronic hepatitis B is significantly correlated with gender and educational level, and females with a low level of education are more likely to develop anxiety (Banerjee et al., 2020). However, the results of the present study revealed that gender, age, occupation, and level of education were not significantly correlated with the incidence of anxiety.

In theory, women are more likely to suffer from anxiety, and patients with higher levels of education may find it easier to understand the occurrence and development of diseases, which should decrease the likelihood that they suffer from anxiety. However, due to the low awareness of DILI in all patients, these factors did not significantly impact the incidence of anxiety. One reason for this result could be the small sample. Therefore, this issue requires further investigation. DILI is most often found in patients using Western medicine, traditional Chinese medicine, and healthcare products, but there were no significant differences in anxiety scores and degree of anxiety between these three types of drugs. This suggests that the type of drug used is not one of the main factors affecting anxiety. As a result of the sporadic cases of DILI and the small sample size of cases caused by the same type of drug, a comparison was not conducted to test whether primary diseases affect the incidence of anxiety.

The degree of liver injury was correlated with the degree of anxiety. One reason for this could be that patients can easily understand the degree of liver injury, and therefore, the poorer the liver function, the more severe the patient considers the illness to be, and anxiety may be related to patients worrying about medical expenses and prognosis. Moreover, the difference in anxiety score between the three groups based on the types of liver injury was not statistically significant, while the difference in degree of anxiety was significant. In theory, the classification of liver injury types is difficult for patients to understand and as a result, should have little effect on anxiety. Therefore, this conclusion is worth further discussion and investigation. Further research revealed that anxiety had no significant impact on the length of stay in hospital or the prognosis for the DILI. The reason for this may be that most of the patients with DILI were acute, had mild anxiety, and had a good prognosis. In hindsight, it would have been helpful to compare anxiety scores before and after treatment.

In summary, the preliminary results of this study confirmed that DILI may be related to anxiety to a certain extent. Although the effect of anxiety on DILI has not reached the level of chronic liver disease, it does suggest that attention should be paid to psychological counseling and education of patients with DILI. In clinical practice, psychological counseling is needed to reduce the anxiety of patients and therefore help them to recover. Due to the sample size and other factors, this study has some limitations, and the conclusions need further confirmation. The sample size should be increased for further in-depth study.

## Data Availability Statement

The original contributions presented in this study are included in the article/supplementary material, further inquiries can be directed to the corresponding author.

## Ethics Statement

The studies involving human participants were reviewed and approved by the Ethics Committee of Hangzhou Red Cross Hospital. The patients/participants provided their written informed consent to participate in this study.

## Author Contributions

Y-HL was involved in drafting the manuscript and revising it critically for important intellectual content, made substantial contributions to conception and design of the work. YG, HX, HF, and D-YC made substantial contributions to the acquisition, analysis, and interpretation of data for the work. All authors read and approved the final manuscript.

## Conflict of Interest

The authors declare that the research was conducted in the absence of any commercial or financial relationships that could be construed as a potential conflict of interest.

## Publisher’s Note

All claims expressed in this article are solely those of the authors and do not necessarily represent those of their affiliated organizations, or those of the publisher, the editors and the reviewers. Any product that may be evaluated in this article, or claim that may be made by its manufacturer, is not guaranteed or endorsed by the publisher.
